# Effects of Two Different Dietary Patterns on Inflammatory Markers, Advanced Glycation End Products and Lipids in Subjects without Type 2 Diabetes: A Randomised Crossover Study

**DOI:** 10.3390/nu9040336

**Published:** 2017-03-29

**Authors:** Yoona Kim, Jennifer B. Keogh, Peter M. Clifton

**Affiliations:** School of Pharmacy and Medical Sciences, University of South Australia, Adelaide SA 5001, Australia; yoona.kim@mymail.unisa.edu.au (Y.K.); Jennifer.Keogh@unisa.edu.au (J.B.K.)

**Keywords:** dietary pattern, plasminogen activator inhibitor type 1, advanced glycation products, insulin sensitivity

## Abstract

Epidemiological studies suggest that consumption of red and processed meat and refined grains are associated with type 2 diabetes and metabolic syndrome and increased inflammatory and fibrinolytic markers. We hypothesised that a diet high in red and processed meat and refined grains (HMD) would increase inflammatory markers and advanced glycation end products (AGEs) compared with a diet high in dairy, whole grains, nuts and legumes (HWD). We performed a randomised crossover study of two four-week interventions in 51 participants without type 2 diabetes (15 men and 36 women aged 35.1 ± 15.6 years; body mass index: 27.7 ± 6.9 kg/m^2^). No baseline measurements were performed. Plasma fluorescent AGEs, carboxymethyllysine, glucose, insulin, lipids, hs-CRP, interleukin 6 (IL-6) and plasminogen activator inhibitor-1 (PAI-1) were analysed after four weeks on each diet. IL-6, hs-CRP, AGEs and carboxymethyllysine were not different between diets but PAI-1 was higher after the HMD than after HWD ((median and interquartile range) 158, 81 vs. 121, 53 ng/mL *p* < 0.001). PAI-1 on the HWD diet was inversely correlated with whole grains intake (*p* = 0.007). PAI-1 was inversely correlated with insulin sensitivity index (r = −0.45; *p* = 0.001) and positively correlated with serum total cholesterol (r = 0.35; *p* = 0.012) and serum triglyceride (r = 0.32; *p* = 0.021) on HMD. This trial was registered with the Australian New Zealand Clinical Trials Registry (ACTRN12614000519651).

## 1. Introduction

Inflammation is thought to play a role in the pathogenesis of type 2 diabetes mellitus (T2DM) [1,2]. Increased concentrations of interleukin 6 (IL-6) and high-sensitivity C-reactive protein (hs-CRP) have been associated with an increased risk of T2DM [3,4]. Elevated plasminogen activator inhibitor type 1 (PAI-1) has also been found to be a predictor of the development of T2DM [5,6,7,8].

Dietary intake may affect inflammatory and fibrinolytic markers. In cross-sectional data obtained from the Insulin Resistance Atherosclerosis Study (IRAS), whole grain consumption was inversely associated with plasma PAI-1 and hs-CRP concentrations and refined grain consumption was positively associated with plasma PAI-1 concentrations [9]. However, a diet high in whole grains for six weeks showed no differences in either hs-CRP or IL-6 or PAI-1 concentrations compared with a diet high in refined grains [10], although reducing the glycemic index of a test food appears to lower PAI-1 [11]. Cross-sectional studies have shown that greater consumption of red and processed meat was associated with higher circulating hs-CRP concentrations [12,13,14,15], while in dietary interventions, red meat consumption did not change hs-CRP concentrations [16,17].

Advanced glycation end products (AGEs) are endogenously formed when the carbonyl groups of reducing sugars and reactive aldehydes non-enzymatically react with the free amino groups in proteins, lipids and nucleic acids through a series of reactions forming Schiff base and Amadori products [18,19,20]. The process of AGEs formation is also known as the Maillard reaction [21]. Higher levels of AGEs are produced in foods processed at high temperatures in dry conditions such as grilling, broiling, frying and roasting, compared with foods processed slowly at lower temperatures or in water [22]. The stable, relatively inert and non-fluorescent carboxymethyllysine (CML) is a frequently measured AGE [20]. High levels of AGEs have been implicated in insulin resistance in non-obese, non-diabetic subjects [23,24] and in pancreatic beta cell dysfunction in vitro and in vivo [25,26]. Dietary AGEs-restricted diets are associated with a lower risk of T2DM [27] and a reduction in inflammation and insulin resistance [28,29].

To date, few interventions have investigated the associations between dietary patterns and inflammatory and fibrinolytic markers and advanced glycation end products in subjects without type 2 diabetes.

We reported on the changes in insulin sensitivity by continuous low-dose insulin and glucose infusion tests (LDIGIT) in our previous publication [30], where we found that the participants fell into an insulin-sensitive group (insulin < 56 pmol/L, *n* = 24), and a relatively insulin-resistant group (insulin > 56 pmol/L, *n* = 25) in response to the LDIGIT _(120–150 min)_ on a diet high in red and processed meat and refined grains (HMD). The insulin-resistant group had significantly higher concentrations of insulin and glucose during the LDIGIT _(120–150 min)_ on the HMD and thus a significantly decreased insulin sensitivity index (ISI), compared with a diet high in dairy, whole grains, nuts and legumes (HWD). In this study, we examined inflammatory and advanced glycation end products that may mediate the insulin resistance, as well as fibrinolytic markers which may be altered by increased insulin resistance. In the previous publication [30], we did not report on any potential mediators of insulin resistance. The specific aim was to determine the effect of a weight-stable diet high in red and processed meat and refined grains compared with a diet high in dairy, whole grains, nuts, legumes without red and processed meat on inflammatory and fibrinolytic markers and advanced glycation end products in individuals without T2DM. It also aimed to examine the association between these markers and the changes in insulin sensitivity found in our previous study [30]. Our hypothesis was that a diet high in red and processed meat and refined grains would elevate concentrations of inflammatory markers (hs-CRP, IL-6), PAI-1 and AGEs (fluorescent AGEs, CML) compared with a diet high in dairy, whole grains, nuts and legumes.

## 2. Methods

### 2.1. Ethical Approval and Registration

This study was approved by the University of South Australia Human Research Ethics committee, and all study participants gave their written informed consent prior to participating. The trial was registered with the Australian New Zealand Clinical Trials Registry www.anzctr.org.au/ (ACTRN12614000519651). AUD$240 was offered to the participants on completion of the two diets. Recruitment began in June 2014 and ended in September 2015.

### 2.2. Study Participants

A total of 51 participants (body mass index (BMI) 18–45 kg/m^2^), aged over 18 years, were recruited by public advertisement and a recruiting agency. An initial screening questionnaire was undertaken via email or telephone. Details of all inclusion, exclusion criteria and the change in entry criteria have been reported previously [30]. Respondents who passed the screening criteria visited the Sansom Institute for Health Research Clinical Trial facility at the University of South Australia, in the morning after an overnight fast. Written informed consent was obtained. Blood pressure was measured four times after sitting quietly for 10 min (Omron Corporation, Kyoto, Japan). The interval between blood pressure measurements was at least 1 min. Height was measured on a wall-mounted stadiometer (Seca, Hamburg, Germany) without shoes and body weight was measured on electronic digital scales (Tanita Corporation, Tokyo, Japan) in light clothing and without shoes. Body composition was measured by whole-body dual-energy X-ray absorptiometry (DXA) (Luna Prodigy, Lunar Radiation Corp., Madison, WI, USA) to calculate lean mass. A 75 g oral glucose tolerance test (OGTT) was performed to exclude people with diabetes. A simple survey on activity levels was carried out at baseline (sedentary or moderately active). We did not collect blood samples at baseline.

A detailed description of participant recruitment and completion has been previously published [30,31].

### 2.3. Dietary Intervention

Eligible participants were randomly allocated to either a diet high in red and processed meat and refined grains (HMD) or a diet high in whole grains, nuts, legumes, dairy, and devoid of red and processed meat (HWD) for four weeks, and then were crossed over to the alternative diet for four weeks. Randomisation was performed via an online random number generator (www.randomization.com) by a researcher not involved in this study.

The investigator who performed data analysis and staff who measured blood samples were blinded to the diet order. There was a minimum of a two-week washout period (on average three weeks) between the two diets during which participants returned to their usual diet. On HMD daily consumption of 200–300 g red meat, ≥50 g processed meat (ham, bacon and sausage) was recommended as this reflected the amount consumed in the highest quintile of published cohort studies [28,32,33]. In order to maximise the difference between the two diets, 4–6 serves of refined grains (white bread, rice, noodles and pasta) based on volunteers’ weights, 1–2 serves of vegetables, 1–2 serves of fruits and a minimal amount of dairy products (e.g., 1–2 teaspoons of milk) were allowed during the HMD. Two hundred to three hundred grams of potatoes, 1 serve of jam or marmalade, 3–9 serves of oil (e.g., olive or canola) or spread (e.g., butter) or 3–4 serves of indulgence food were recommended for additional energy in accordance with volunteers’ weights. During the HWD, recommendations, based on the volunteers’ weights, were daily consumption of 4 serves of low-fat dairy products including 2 serves of yoghurt, 3–4 serves of whole grain breads, rice and pasta, 60–90 g unsalted nuts and either 70–150 g chicken or fish (or other seafood) or 150–225 g cooked legumes. Serve sizes of cereal foods were as follows: 1 slice (40 g) wholegrain or wholemeal bread, ½ cup (75–120 g) cooked brown rice, ½ cup (75–120 g) cooked wholemeal pasta, ¼ cup (30 g) muesli, ½ cup oats, ⅔ cup (30 g) wheat cereal flakes, ½ cup (120 g) cooked wholegrain porridge, 3 (35 g) wholegrain crisp-breads. Two to seven serves of oil or spread and one serve of jam or marmalade were also advised for additional energy intake in accordance with volunteers’ weights. The recommended menus for each of the diets have been published previously [31].

Total energy was matched in the two diets. Vegetables and fruits were limited to 1–2 serves in both diets as epidemiology does not suggest that these components are related to the risk of type 2 diabetes [34,35]. Serving sizes were defined according to the guidelines of the Australian National Health and Medical Research Council [36]. Food was not provided to participants but they were given an AUD$80 voucher for red meat and an AUD$50 voucher for the nuts at the end of each diet period. Participants received specific dietary instructions for each diet along with eight different energy levels based on BMI and gender. For the HWD, healthy cooking methods included boiling, steaming, stewing and poaching rather than deep-frying, grilling, and roasting. Samples of recipes and daily meal plans for the two diets were given. A kitchen scale (Homemaker Slimline Electronic Scale; Kmart, Melbourne, Australia) was provided to measure the quantity of recommended food at home. Participants were encouraged to check their weight every day and maintain usual physical activity. Weight was measured at each visit and in the event of weight change, the diet was adjusted accordingly. A daily checklist during each dietary period and a three-day weighed food diary specifying serving weights and the types of food they consumed within each two-week period were used in order to monitor dietary compliance. Participants attended the clinic fortnightly and the three-day weighed food diary and daily checklists were checked. All food consumed over these three-day weighed periods were analysed by Food Works Professional Edition 8.0 (Xyris, Kenmore Hills, Qld, Australia). Dietary intake was not measured before the intervention, at baseline or during the washout period.

### 2.4. Assessments of Insulin Sensitivity

At the end of each diet period, participants attended the Sansom Institute for Health Research Clinical Trial facility from 8 a.m. to 9 a.m. in the morning after an overnight fast for a continuous low-dose insulin and glucose infusion test ((LDIGIT); *n* = 49). All fasting blood samples were taken at this time and individuals had the same appointment time for each visit. Fifty-one participants completed the study but only 49 subjects could be included due to failure of blood sample collections or cannulations for the low-dose insulin and glucose infusion test (LDIGIT). If volunteers were sick prior to the day of the test, we deferred the test till they were well again. The dietary intervention continued until all blood sampling was completed. All medications changes were recorded.

Descriptions of the LDIGIT administration and calculation of ISI and homeostasis model assessment of insulin resistance (HOMA-IR) have been previously reported [30].

During the tests, blood for serum was collected in the tube with no additives and the tube was kept upright in a tube rack at room temperature for 30 min to ensure complete clot formation and then placed on ice. Blood for plasma was collected in the sodium fluoride EDTA tube and the tube was placed immediately on ice until centrifugation and processing. Blood samples were centrifuged at 4000 RPM at 4 °C for 10 min (Universal 32R, Hettich Zentrifugen, Tuttlingen, Germany).

### 2.5. Biomarkers Analysis

Plasma glucose, fasting serum total cholesterol, triglyceride (TG), high-density lipoprotein cholesterol (HDL-C) and hs-CRP were measured using an automated spectrophotometric analyzer (Konelab 20XTi, Thermo Electron, Massachusetts, MA, USA). Serum insulin was assayed by using a commercial ELISA kit (Alpha Diagnostic, San Antonio, TX, USA, Kit # 0030N) with an intra-assay coefficient of variation (CV) of 6.29%–10.40% and inter-assay CV of 5.8%–11.8%. IL-6 was assayed by using a commercial ELISA kit (Elisakit.com, Victoria, Australia, #: 0012 Human IL-6 ELISA Kit) with an intra-assay CV of <10% and inter-assay CV of <10%. PAI-1 was assayed by using a commercial ELISA kit (Affymetrix eBioscience, San Diego, CA, USA, BMS2033/BMS2033TEN Human PAI-1 Platinum ELISA kit) with an intra-assay CV of 4.7% and inter-assay CV of 5.0%. Serum CML was measured by using a competitive ELISA kit (CircuLex, Nagano, Japan, CML/Nε (Carboxymethyl) lysine ELISA Kit, Cat# CY-8066) with an intra-assay CV of 5.2%–7.4% and inter-assay CV of 4.7%–15.2%.

Serum was diluted 50-fold with phosphate-buffered saline and 250 µL of the diluted serum was aliquoted into the black 96-well plate in triplicate. Total fluorescent AGEs were measured in a multi-mode microplate reader (EnSpire^®^ Multimode Plate Reader, PerkinElmer, Waltham, MA, USA) at room temperature to estimate the levels of fluorescent AGEs. Fluorescence was read at excitation and emission wavelengths of 370 nm and 440 nm, respectively.

### 2.6. Statistical Analyses

Data were analyzed with SPSS V22 (IBM, Chicago, IL, USA). The power calculation was based on our primary outcome of insulin sensitivity with a sample size of 50 overweight and obese subjects without diabetes providing 80% power to detect a 20% change (alpha level = 0.05) in insulin sensitivity as assessed by LDIGIT [37]. The Shapiro–Wilk test, Q–Q plots, and histograms were used to test for the normality of distribution. Non-normally distributed variables were log transformed before analysis but untransformed data is shown. Differences between diets were tested by paired samples *t*-tests. Wilcoxon signed-rank nonparametric tests were also used as some variables (e.g., fasting glucose, fasting insulin, HOMA-IR, hs-CRP, PAI-1 and IL-6) were still skewed in one or both diet periods after log transformation. Associations were examined using Pearson correlation coefficients for parametric correlations, and Spearman correlation coefficients and Kendall’s tau for non-parametric correlations.

An unpaired *t*-test, a chi-square test and a Mann–Whitney nonparametric test were performed to contrast variables between post hoc insulin sensitivity groups. *p* values for group by time interaction were determined by repeated measures of ANOVA. Data are presented as the means ± standard deviations (SDs) except for skewed variables which are expressed as medians and interquartile ranges. Statistical significance was defined as *p* < 0.05.

## 3. Results

### 3.1. Study Participants

Fifty-one participants completed the two diets and forty-nine participants underwent a successful LDIGIT. Table 1 shows the baseline characteristics of a total of 51 participants divided into 2 LDIGIT groups (a total of 49 participants) defined a posteriori based on insulin values during the LDIGIT_120–150 min_, not from randomised groups: an insulin-sensitive group <56 pmol/L with a median insulin of 33 pmol/L (*n* = 24) and an insulin-resistant group >56 pmol/L with a median insulin of 122 pmol/L (*n* = 25).

Body weight remained very stable throughout the study period. The average weights were 77.9, 77.8 and 77.9 kg during the HMD period (baseline, 2 w and 4 w) and 77.8, 77.7 and 77.8 kg during the HWD period. In the LDIGIT insulin-resistant group, insulin (*p* = 0.019) and glucose (*p* = 0.05) were higher after HMD than after HWD, resulting in a decreased ISI (*p* = 0.014). Log ISI HMD was correlated with BMI (*p* = 0.009) and fat mass (*p* = 0.004). Log ISI HWD was positively correlated with the amount of carbohydrates in the HWD (Beta-coefficient 0.27; t = 3.5; *p* = 0.001) after adjustment for log ISI HMD (Beta-coefficient 0.79; t = 10.3; *p* < 0.001).

### 3.2. Dietary Compliances and Intakes

The reported dietary intake of nutrients and key foods assessed by weighed food records (*n* = 51) during the two diet periods has been previously published [31]. Analyses of three-day weighed food diaries of every two weeks (a total of six-day weighed food diaries for each four-week dietary period) indicated that dietary compliance was good with similar energy intake in the two diets, with intakes of key foods very similar to the recommended quantities of key foods. We achieved our planned differences in red and processed meat and whole and refined grains. The key foods consumed were 241 g of red meat, 56 g of processed meat (19% protein) and 320 g of refined grains (44% carbohydrate) compared with 70 g of nuts, 236 g of wholegrains and 687 g of dairy foods (19% protein, 37% carbohydrate). We took no biomarkers of food intake as protein intake was planned to be the same on both diets and estimated protein intake was not different at 95 and 97 g/day while total fat and carbohydrate, which were a little different, have no agreed sensitive biomarkers other than HDL cholesterol and triglyceride, which were measured. Fat energy was 6% higher and carbohydrate 7% lower on the HWD.

### 3.3. Inflammatory Markers

Table 2 summarises the differences in insulin sensitivity, lipids, inflammatory and fibrinolytic markers, and AGEs between the two diets measured at the end of each dietary period in 51 participants. There were no confounding illnesses or medications or smoking at the time of the blood sampling and the one person who felt unwell at the time of their appointment was rescheduled for three days later. No medication was taken. Diet order had no effect in either LDIGIT group. PAI-1 was significantly higher after HMD than after HWD (*p* < 0.001; *n* = 51). PAI-1 HWD was inversely correlated with the log of whole grains intake (r = 0.29; *p* = 0.04; *n* = 51). There was a strong relationship on linear regression between PAI-1 HWD and whole grains intake (Beta-coefficient = −0.35; t = −2.8; *p* = 0.007) and PAI-1 HMD (*p* = 0.001).

PAI-1 HMD was inversely correlated with log ISI _LDIGIT_ after HMD (r = −0.45; *p* = 0.001, *n* = 49). When two groups were defined a posteriori based on the insulin values of LDIGIT_(120–150 min)_, there was a borderline significant group by time interaction for PAI-1 (*p* = 0.05; *n* = 49) and the change in PAI-1 was greater in the insulin-resistant group (177, 61 for HMD vs. 128, 52 ng/mL for HWD; *p* < 0.001) than in the insulin-sensitive group (137, 80 for HMD vs. 113, 39 ng/mL for HWD; *p* = 0.02). There was no effect of diet order on these results nor was there a difference between glycemic control groups [normal compared with impaired fasting glucose (IFG)/impaired glucose tolerance (IGT)].

PAI-1 HMD was positively correlated with serum total cholesterol on the HMD (r = 0.35; *p* = 0.01; *n* = 51) and with log serum TG after the HMD (r = 0.32; *p* = 0.02; *n* = 51).

There were no differences in hs-CRP (*p* = 0.7; *n* = 51) and IL-6 (*p* = 0.7; *n* = 51) between the two diets.

### 3.4. Advanced Glycation end Products

Total fluorescent AGEs were not different between the diets. Fluorescent AGEs after the HWD were correlated with dietary sugars (r = 0.3; *p* = 0.04; *n* = 51). CML did not differ between the two diets (*p* = 0.07; *n* = 51). Glycemic status (normal compared with IFG/IGT) had no effect.

### 3.5. Lipids

Total cholesterol was significantly higher after HMD than after HWD (*p* = 0.03; *n* = 51). HDL-C did not differ between the two diets (*p* = 0.4; *n* = 51). Triglyceride was significantly higher after HMD than after HWD (*p* = 0.04; *n* = 51). Glycemic status (normal compared with IFG/IGT) had no effect.

## 4. Discussion

The present weight-stable randomised crossover study showed that the consumption of a high red and processed meat and a low-fibre, medium glycemic index (GI) and medium glycemic load (GL) diet for four weeks significantly increased PAI-1 concentrations compared with consumption of a high dairy, whole grains, nuts and legumes low GI and low glycemic load diet. In addition, there was a significant LDIGIT group by time interaction for the PAI-1, showing that this effect was more marked in the insulin-resistant group. This finding strongly supports our previous conclusion that an increase in whole grain foods in the HWD rather than the source of protein and the type of fat was related to an increase in insulin sensitivity in only relatively insulin-resistant subjects.

Our finding of PAI-1 being inversely correlated with whole grains intake is consistent with the finding that refined grains intake was positively, and whole grains intake inversely, associated with PAI-1 concentrations in a cross-sectional study in the nondiabetic population [9].

Our finding is consistent with other randomised crossover [11,38,39] and parallel [40] grain interventions. These studies show that an oat bran diet (102 g/day of oat bran added to a low-fiber diet) for two weeks lowered PAI-1 concentrations compared with a low-fiber diet in 24 young healthy subjects [38] and a low-GI diet reduced PAI-1 concentrations in 20 patients with T2DM [11], 12 men with T2DM [39] and 50 overweight women [40]. However, the data is quite mixed and there are studies showing that a whole grain diet in subjects with normal or impaired carbohydrate metabolism [41], obese subjects with metabolic syndrome [42] and healthy, overweight subjects [10] had no effect on PAI-1 compared with a refined grain diet.

PAI-1, a single-chain glycoprotein (47 kilodaltons), is an inhibitor of tissue plasminogen activator (tPA) [43]. PAI-1 plays a role in thrombus formation and cardiovascular disease [43,44]. The Insulin Resistance Atherosclerosis Study suggested that elevated PAI-1 concentrations might be a very early risk marker of the insulin resistance syndrome leading to T2DM [6]. Our finding that PAI-1 was inversely correlated with insulin sensitivity in the HMD strongly supports this suggestion.

Moreover, our current study found that the HMD significantly increased serum total cholesterol and triglyceride compared with the HWD. Positive correlations between PAI-1 and serum cholesterol and triglyceride were observed. These correlations suggest that PAI-1 is closely allied with other risk factors for atherosclerosis [45]. An intervention [38] showing a decrease in both PAI-1 and lipid profiles (total cholesterol, HDL-C and low-density lipoprotein cholesterol (LDL-C)) after an oat bran diet, however, failed to find the association between the changes in PAI-1 and lipid profiles. Two interventions [11,39] which showed favourable effects of a low-GI diet on insulin sensitivity, lipid profiles and PAI-1 in subjects with T2DM compared with a high-GI diet, however, did not find an association between PAI-1 and insulin sensitivity or lipid profiles. Therefore, our study is the first dietary intervention observing correlations between PAI-1, lipid profiles (total cholesterol and triglyceride) and insulin resistance as previously shown in the cross-sectional studies.

In this study, we could not show differences in CML levels between a diet high in red and processed meat and refined grains compared with a diet high in whole grain, legumes, nuts and dairy products. A wide range of foods in a modern diet contains AGEs [46,47]. Uribarri et al. [46] reported that the highest dietary CML levels were observed in beef and cheese, followed by poultry, pork and fish. CML contents in roasted nuts (peanuts, walnuts, almonds and cashews) range from 6447 to 9807 kU/100 g, which are the almost same amount of AGEs in 100 g of beef steak strips, stir-fried with or without oil from 7 min to 15 min [46]. Even though red and processed meat and white bread are rich sources of dietary CML, cooking methods could drive CML formation in the HWD diet. For example, the AGE content of boiled or stewed chicken (1124 kU/100 g) increases when the same amount of chicken is broiled (5828 kU/100 g). The AGE content of broiled chicken is similar to the AGE content of broiled beef (5963 kU/100 g) while the AGE content of boiled or stewed beef is 2230 kU/100 g [46]. Tessier et al. [21] reported that breakfast cereal and dairy products accounted for 12% and 4% of the entire dietary intake of CML [21]. Cheese and grilled fish (e.g., salmon ranging from 3000 to 4300 kU/100 g) also contribute to dietary intake of CML [46].

In light of the suggestion that foods rich in protein and fat are more likely to have a higher AGEs content [48], a similar intake of protein and saturated fat in the HMD and HWD should mean little potential difference in CML contents between the two diets. In addition, there was a significantly higher intake of vitamin C (an antioxidant involved in inhibiting AGE formation [19]) from potato in the HMD which cannot be ignored as CML is known to be a glyoxidation product [19,20]. It has been proposed that dietary AGEs are associated with generation of pro-inflammatory cytokines in healthy subjects [49]. In our study, no differences in the inflammatory molecules hs-CRP and IL-6 between the two diets may be explained by the absence of a difference in CML levels between the two diets. The absence of differences in hs-CRP and IL-6 at the end of the two interventions could be partly attributed to the low inflammatory milieu of the study sample.

Recent evidence has challenged the concept that IL-6 has a negative impact on metabolic homeostasis [50,51]. The further measurements of IL-1beta/IL-1ra or TNF alpha which are associated with insulin resistance [52] may be useful. However, these measurements were not undertaken in this study.

The strength of this study is that, to our knowledge, this is the first randomised crossover intervention in weight stability showing significantly higher PAI-1 concentrations after the consumption of red and processed meat and refined grains than after the consumption of whole grains, nuts, dairy products and legumes. This is also the first dietary intervention showing a correlation between PAI-1, insulin resistance and lipid profile changes. The crossover and isocaloric study design with tightly controlled weight and activity levels is a strength of this study.

There are several limitations to take into account when the results are interpreted. We did not measure faecal and urinary CML. Studies show that only about 10%–30% of dietary AGEs are absorbed in the intestine and one-third of ingested AGEs are excreted in urine and faeces, and two-thirds are retained in tissues [22,53,54]. We did not have baseline measurements for any variable. Meal preparation and consumption outside the clinical research facility could be a potential limitation, although we believe compliance to the diets was excellent. The other potential limitation could be that dietary intake was not measured before the intervention, at baseline or during the washout and we had no control group, nor did we have objective measures of nutrient intake.

In conclusion, our study suggests that a diet high in red and processed meat and refined grains for four weeks significantly elevated PAI-1 concentrations compared with a diet high in whole grains, nuts, dairy products and legumes. Increased PAI-1 may be associated with insulin resistance and elevated lipids. This study provides more evidence in favour of a healthy diet pattern for disease prevention and that higher-quality fibre-rich carbohydrate may be the most important element in this healthy diet.

## Figures and Tables

**Table 1:** Baseline characteristics of participants.

Variable	All Participants ^1^	Participants Who Completed LDIGIT (*n* = 49)		
		Insulin-Sensitive Group (*n* = 24)	Insulin-Resistant Group (*n* = 25)	*p*
Sex (M/F)	15/36	9/15	6/19	0.3 ^†^
Age (year)	35.1 ± 15.6	35.5 ± 15.6	35.6 ± 16	1
NGT (*n*)	17	10	6	0.2
IFG/IGT (*n*)	34	14	19	
Baseline fasting glucose (mmol/L)	5.5 ± 0.7	5.3 ± 0.8	5.7 ± 0.6	0.07
Baseline 2 h glucose (mmol/L)	7.28 ± 1.6	6.96 ± 1.5	7.55 ± 1.7	0.2
HOMA-IR (HWD)	0.37, 0.43	0.29, 0.47	0.39, 0.5	0.07 ^‡^
HOMA-IR (HMD)	0.53, 0.66	0.21, 0.54	0.65, 0.8	0.01 ^‡^
LDIGIT_(120–150 min)_ insulin (HWD pmol/L) ^2^		33.4, 27.8	122.7, 149	<0.001 ^#^
LDIGIT_(120–150 min)_ insulin (HMD pmol/L) ^2^		29.6, 20.3	153, 180	<0.001 ^#^
Baseline weight (kg)	79.4 ± 21.36	69.7 ± 15.1	85.7 ± 21.7	0.005
BMI (kg/m^2^)	27.7 ± 6.9	24.4 ± 4.6	29.4 ± 5.9	0.002
Baseline SBP (mmHg)	112.2 ± 10.7 ^3^	110.4 ± 9.2 ^4^	114.3 ± 12.4 ^5^	0.3
Baseline DBP (mmHg)	70.7 ± 9.7 ^3^	69.1 ± 9.9 ^4^	72.5 ± 9.5 ^5^	0.3
Total Fat Mass (kg)	29 ± 15.7	20.6 ± 11.4	34.3 ± 13.3	<0.001
Total Lean Mass (kg)	46.6 ± 11.5	45.4 ± 11.4	47.7 ± 12.1	0.5
Total Fat Mass (%)	36.6 ± 12.6	30.3 ± 12.8	41.0 ± 8.6	0.001

Groups were contrasted with an unpaired *t*-test. ^†^ *p* values were obtained by chi-square test. ^‡^ *p* values were obtained by Mann–Whitney nonparametric test. ^#^ *p* values were obtained by unpaired *t*-tests after log transformation. Values are means ± SDs except for HOMA-IR and LDIGIT_(120–150 min)_ insulin, which are medians and interquartile ranges. The insulin-sensitive group and insulin-resistant group were defined a posteriori based on the insulin values of LDIGIT_120–150 min_, not from randomised groups: insulin-sensitive group <56 pmol/L with a median insulin of 33 pmol/L (*n* = 24) and insulin-resistant group >56 pmol/L with a median insulin of 122 pmol/L (*n* = 25). M, male; F, female; NGT, normal glucose tolerance; IFG, impaired fasting glucose; IGT, impaired glucose tolerance; BMI, body mass index; SBP, systolic blood pressure; DBP, diastolic blood pressure; LDIGIT, low-dose insulin and glucose infusion tests; HOMA-IR, homeostasis model assessment of insulin resistance; HWD, high in dairy, whole grains, nuts and legumes; HMD, high in red and processed meat and refined grains; SD, standard deviation. ^1^ *n* = 51; ^2^ *n* = 49, ^3^ *n* = 42; ^4^ *n* = 22; ^5^ *n* = 19.

**Table 2:** Insulin sensitivity, lipids, inflammatory and fibrinolytic markers, and AGEs measured at the end of each dietary period in all participants (*n* = 51).

Variable	HMD	HWD	*p*
**Fasting glucose (mmol/L)**	5.3, 0.6	5.3, 0.5	0.9
**Fasting insulin (pmol/L)**	11.8, 15	9, 11	0.3
**HOMA-IR**	0.53, 0.66	0.37, 0.43	0.25
**TG (mmol/L)**	0.91, 0.72	0.86, 0.69	0.041
**HDL-C (mmol/L)**	1.39 ± 0.43	1.37 ± 0.42	0.4
**Total cholesterol (mmol/L)**	4.8 ± 1.0	4.6 ± 1.0	0.032
**hs-CRP (mg/L)**	1.01, 2.98	0.61, 2.55	0.7
**IL-6 (pg/mL)**	8, 12	8, 12	0.65
**PAI-1 (ng/mL)**	158, 81	121, 53	<0.001
**Fluorescent AGEs (absorbance units)**	1388, 304	1370, 454	0.16
**CML (µg/mL)**	1.37, 0.67	1.49, 0.56	0.07

*p* values (*n* = 51) for fluorescent AGEs, TG, CML, total cholesterol and HDL-C were determined by paired *t*-tests. Fluorescent AGEs, TG and CML were log transformed. *p* values (*n* = 51) for fasting glucose, fasting insulin, HOMA-IR, hs-CRP, PAI-1 and IL-6 were obtained from nonparametric tests. Values (*n* = 51) are expressed as medians and interquartile ranges except for HDL-C and total cholesterol, which are presented as means ± SDs. HMD, a high red and processed meat and refined grains diet; HWD, a high whole grain, nuts, dairy and legumes diet; HOMA-IR, homeostasis model assessment of insulin resistance; TG, triglyceride; HDL-C, high density lipoprotein cholesterol; hs-CRP, high sensitivity C-reactive protein; IL-6, interleukin 6; PAI-1, plasminogen activator inhibitor type 1; AGE, advanced glycation end product; CML, carboxymethyllysine.

## Fundin

Yoona Kim is supported by an Australian Government Research Training Program Scholarship. Peter M. Clifton is supported by a National Health and Medical Research Council Principal Research Fellowship.
